# The PedsQL™ Family Impact Module: Preliminary reliability and validity

**DOI:** 10.1186/1477-7525-2-55

**Published:** 2004-09-27

**Authors:** James W Varni, Sandra A Sherman, Tasha M Burwinkle, Paige E Dickinson, Pamela Dixon

**Affiliations:** 1 Department of Landscape Architecture and Urban Planning, College of Architecture, Texas A&M University, 3137 TAMU, College Station, TX 77843-3137 USA; 2 San Diego State University/ University of California, San Diego Joint Doctoral Program in Clinical Psychology, 6363 Alvarado Court, Suite 103, San Diego, CA 92120 USA; 3 Department of Anesthesiology, University of Washington, 1945 NE Pacific Street, Seattle, WA 98195 USA; 4 California School of Professional Psychology, 10455 Pomerado Rd., San Diego, CA 92131 USA; 5 Children's Convalescent Hospital, Children's Hospital and Health Center, 3020 Children's Way, San Diego, CA 92123 USA

**Keywords:** health-related quality of life, family, pediatrics, children, PedsQL™

## Abstract

**Background:**

The PedsQL™ Measurement Model was designed to measure health-related quality of life (HRQOL) in children and adolescents. The PedsQL™ 4.0 Generic Core Scales were developed to be integrated with the PedsQL™ Disease-Specific Modules. The newly developed PedsQL™ Family Impact Module was designed to measure the impact of pediatric chronic health conditions on parents and the family. The PedsQL™ Family Impact Module measures parent self-reported physical, emotional, social, and cognitive functioning, communication, and worry. The Module also measures parent-reported family daily activities and family relationships.

**Methods:**

The 36-item PedsQL™ Family Impact Module was administered to 23 families of medically fragile children with complex chronic health conditions who either resided in a long-term care convalescent hospital or resided at home with their families.

**Results:**

Internal consistency reliability was demonstrated for the PedsQL™ Family Impact Module Total Scale Score (α = 0.97), Parent HRQOL Summary Score (α = 0.96), Family Functioning Summary Score (α = 0.90), and Module Scales (average α = 0.90, range = 0.82 – 0.97). The PedsQL™ Family Impact Module distinguished between families with children in a long-term care facility and families whose children resided at home.

**Conclusions:**

The results demonstrate the preliminary reliability and validity of the PedsQL™ Family Impact Module in families with children with complex chronic health conditions. The PedsQL™ Family Impact Module will be further field tested to determine the measurement properties of this new instrument with other pediatric chronic health conditions.

## Background

Pediatric health-related quality of life (HRQOL) is increasingly acknowledged as an important health outcome measure in clinical trials and health services research and evaluation [1,2]. Additionally, in pediatric chronic health conditions, the impact of disease and treatment on family functioning is a salient concern given the essential role of the family in child adaptation to disease [3-5]. Within this context, the impact of pediatric chronic health conditions on the family has been conceptualized within a theoretical risk and resistance framework, in which parent adjustment and the family system as a whole have been identified at increased risk [6].

Although there are a number of well-developed generic measures of family functioning, such as the Family Environment Scale [7], instruments that specifically measure the impact of pediatric chronic health conditions on parent and family functioning are less common. The two most widely utilized family impact instruments are the Impact on Family Scale and the Child Health Questionnaire (CHQ). The Impact on Family Scale-Revised is a brief unidimensional instrument that measures one factor of general negative impact on the social and familial systems and has demonstrated good reliability and validity in the samples tested [8]. The CHQ, a well validated instrument which contains scales measuring child HRQOL [9], contains a scale measuring whether the child's health or behavior limited family activities or caused family conflict. The CHQ also contains two parent self-report scales which measure the impact of the child's health on parent worry or concern and limitations in meeting their own needs.

Although these two well-developed measures existed when we conceptualized the PedsQL™ Family Impact Module, after an analysis of the items and scales of the existing instruments, we felt that a PedsQL™ Family Impact Module would make a significant contribution to the literature by creating a multidimensional instrument that could stand alone, or be easily integrated into the PedsQL™ Measurement Model [10]. The PedsQL™ Measurement Model includes not only generic health-related quality of life [11-13] and disease-specific measurement instruments [14-18], but also generic measures of fatigue [15,19], healthcare satisfaction [20,21] and evaluations of the healthcare built environment [21]. Thus, we envisioned a Family Impact Module that would contribute to the literature by identifying items and scales which were not redundant with existing instruments, and which would further enhance the measurement options available through the PedsQL™ Measurement Model.

In this context, the PedsQL™ Family Impact Module was developed and initially field tested in families with medically fragile children with complex chronic medical conditions as part of our evaluation of the healing environment of a Children's Convalescent Hospital [21]. In order to provide a contrast group to these children in this long-term care facility, we selected a population of children with comparable complex chronic medical conditions who were residing at home with their families. Since these children's severe medical conditions prevented them from providing self-report, the PedsQL™ Family Impact Module was designed as a parent proxy-report instrument.

This study investigates the preliminary reliability and validity of the PedsQL™ Family Impact Module in medically fragile children with complex chronic health conditions. We hypothesized that the PedsQL™ Family Impact Module would distinguish between families in which the child resided at home versus those whose child resided in a long-term care facility based on the extant literature on pediatric chronic health conditions and the impact on parents and families [5,6].

## Method

### Participants and Settings

Participants were the parents of 23 medically fragile pediatric patients with complex chronic health conditions, such as severe cerebral palsy and birth defects. Participants from the Children's Convalescent Hospital (CCH) were parents of 12 pediatric patients who were residents of this long-term care facility. For each CCH family, the family member who completed the PedsQL™ Family Impact Module was the resident's mother. Participants from the REACH program (an outpatient program designed to reach out to families who choose to take care of their medically fragile children at home) were the parents of 11 pediatric patients. For each REACH family except one, the family member who completed the PedsQL™ was the patient's mother.

### PedsQL™ Family Impact Module

The 36-item PedsQL™ Family Impact Module Scales encompass 6 scales measuring parent self-reported functioning: 1) Physical Functioning (6 items), 2) Emotional Functioning (5 items), 3) Social Functioning (4 items), 4) Cognitive Functioning (5 items), 5) Communication (3 items), 6) Worry (5 items), and 2 scales measuring parent-reported family functioning; 7) Daily Activities (3 items) and 8) Family Relationships (5 items). Items and scales were developed through focus groups, cognitive interviews and pre-testing measurement development protocols [10,11], and our prior research and clinical experiences with children with chronic health conditions and their families. Table 1 contains a general description of the scale items.

**Table 1:** PedsQL™ Family Impact Module – general content of scales

**Parent Functioning**	**# Items**	**General Content**
Physical Functioning	6	Problems with physical functioning, including feeling tired, getting headaches, feeling weak, and stomach problems
Emotional Functioning	5	Problems with emotional functioning, including anxiety, sadness, anger, frustration, and feeling helpless or hopeless
Social Functioning	4	Problems with social functioning, including feeling isolated, difficulty getting support from others, and finding time or energy for social activities
Cognitive Functioning	5	Problems with cognitive functioning, including difficulty maintaining attention, remembering things, and thinking quickly
Communication	3	Problems with communication, including others not understanding the family's situation, difficulty talking about child's health condition, and communicating with health professionals
Worry	5	Problems with worrying, including worrying about child's treatments and side effects, about others' reactions to child's condition, about the effect of the illness on the rest of the family, and about child's future
**Family Functioning**	**# Items**	**General Content**

Daily Activities	3	Problems with daily activities, including activities taking more time and effort, difficulty finding time and energy to finish household tasks
Family Relationships	5	Problems with family relationships, including communication, stress, and conflicts between family members, and difficulty making decisions and solving problems as a family

Total Score is computed by averaging all 36 items. Parent HRQOL Summary Score is computed by averaging 20 items in Physical, Emotional, Social, and Cognitive Functioning. Family Summary Score is computed by averaging 8 items in Daily Activities and Family Relationships.

The PedsQL™ Family Impact Module was developed as a parent-report instrument. A 5-point response scale is utilized (0 = never a problem; 4 = always a problem). Items are reverse-scored and linearly transformed to a 0–100 scale (0 = 100, 1 = 75, 2 = 50, 3 = 25, 4 = 0), so that higher scores indicate better functioning (less negative impact). Scale Scores are computed as the sum of the items divided by the number of items answered (this accounts for missing data). If more than 50% of the items in the scale are missing, the Scale Score is not computed [22]. Although there are other strategies for imputing missing values, this computation is consistent with the previous PedsQL™ peer-reviewed publications, as well as other well-established HRQOL measures [23,24].

The PedsQL Family Impact Module Total Scale Score is the sum of all 36 items divided by the number of items answered. The Parent HRQOL Summary Score (20 items) is computed as the sum of the items divided by the number of items answered in the Physical, Emotional, Social, and Cognitive Functioning Scales. The Family Functioning Summary Score (8 items) is computed as the sum of the items divided by the number of items answered in the Daily Activities and Family Relationships Scales.

### Procedure

The PedsQL™ Family Impact Module was mailed to families whose children were residents at the CCH and outpatients in the REACH program, along with a self-addressed stamped envelope in which to return the survey to the research team. A letter was included in the packet explaining the study, the confidentiality with which their data would be treated, and that the healthcare staff would not see this information. The protocol was approved by the Institutional Review Board at Children's Hospital and Health Center, San Diego.

### Statistical Analysis

Scale internal consistency reliability was determined by calculating Cronbach's coefficient alpha [25]. Scales with reliabilities of 0.70 or greater are recommended for comparing patient groups, while a reliability criterion of 0.90 is recommended for analyzing individual patient scale scores [26,27].

Construct validity for the PedsQL™ Family Impact Module was determined utilizing the known-groups method. The known-groups method compares scale scores across groups known or expected to differ in the construct being investigated. In this study, PedsQL™ Family Impact Module scores in groups differing in residence of the child (Convalescent Hospital inpatient sample versus REACH outpatient sample) were computed [28,29], using independent sample t-tests. We hypothesized that families whose children were residents in the Convalescent Hospital would report significantly higher scores (less negative impact) than families whose children were being taken care of at home based on the exant literature on pediatric chronic health conditions, families, and parental adjustment [6]. In order to determine the magnitude of the differences between families, effect sizes were calculated [30]. Effect size as utilized in these analyses was calculated by taking the difference between the Convalescent Hospital sample mean and the REACH sample mean, divided by the pooled standard deviation. Effect sizes for differences in means are designated as small (.20), medium (.50), and large (.80) in magnitude [30]. Statistical analyses were conducted using SPSS for Windows.

## Results

### Means and Standard Deviations

Table 2 presents the means and standard deviations of the Convalescent Hospital inpatient sample and the REACH outpatient sample.

**Table 2:** Scale descriptives for PedsQL™ Family Impact Module: Comparisons across CCH and REACH samples

		**CCH Sample**			**REACH Sample**				
**Scale**	**# Items**	**N**	**Mean**	**SD**	**N**	**Mean**	**SD**	**Difference**	**Effect Size**

Total Impact Score	36	12	81.00	17.06	11	62.49	17.26	18.51**	1.08
Parent HRQOL Summary	20	12	83.75	15.55	11	62.94	19.83	20.81***	1.17
Physical Functioning	6	12	82.99	17.36	11	53.03	22.83	29.26***	1.45
Emotional Functioning	5	12	78.33	18.26	11	64.48	26.59	13.85	0.61
Social Functioning	4	12	85.42	17.34	11	61.93	25.99	23.49**	1.07
Cognitive Functioning	5	12	88.75	12.81	11	74.09	18.95	14.66*	0.91
Communication	3	12	73.61	24.58	11	52.15	24.67	21.46*	0.87
Worry	5	12	69.17	21.09	11	56.82	25.52	12.35	0.53
Family Summary	8	12	84.27	20.47	11	68.81	24.11	15.46	0.69
Daily Activities	3	12	85.14	24.75	11	51.89	31.48	33.25***	1.18
Family Relationships	5	12	83.75	23.07	11	78.95	27.62	4.80	0.19

Note: Higher values equal better health-related quality of life and family functioning. HRQOL = health-related quality of life; CCH = Children's Convalescent Hospital. REACH = outpatient sample.

*p < .05, **p < .02, ***p < .01; equal variances not assumed. Effect sizes are designated as small (.20), medium (.50), and large (.80).

### Internal Consistency Reliability

Internal consistency reliability alpha coefficients for the PedsQL™ Family Impact Module Scales are presented in Table 3. The scales exceeded the minimum reliability standard of 0.70 [26]. Most PedsQL™ Family Impact Module Scales approached or exceeded the reliability criterion of 0.90 recommended for analyzing individual patient scale scores [26,27].

**Table 3:** PedsQL™ Family Impact Module: Internal consistency reliability for total, CCH, and REACH samples

**Scale**	**Total**	**N**	**CCH**	**N**	**REACH**	**N**
Total Impact Score	.97	23	.97	12	.95	11
Parent HRQOL Summary Score	.96	23	.96	12	.95	11
Physical Functioning	.91	23	.84	12	.88	11
Emotional Functioning	.90	23	.83	12	.93	11
Social Functioning	.88	23	.87	12	.88	11
Cognitive Functioning	.93	23	.93	12	.91	11
Communication	.88	23	.79	12	.95	11
Worry	.82	23	.80	12	.84	11
Family Summary Score	.90	23	.93	12	.89	11
Daily Activities	.91	23	.95	12	.83	11
Family Relationships	.97	23	.98	12	.96	11

Note: HRQOL = health-related quality of life; CCH = Children's Convalescent Hospital. REACH = outpatient sample.

### Construct Validity

Table 2 presents the effect sizes and t-test results of the PedsQL™ Family Impact Module Scales for families with children at the CCH and REACH. The effects sizes were all in the medium to large effect size range except for one scale. Although the small sample size decreases the probability of detecting statistically significant differences, 7 of the 11 comparisons were statistically significant.

## Discussion

This study presents the preliminary reliability and validity of the newly developed PedsQL™ Family Impact Module. All internal consistency reliabilities exceeded the recommended minimum alpha coefficient standard of 0.70 for group comparisons, with most scales approaching or exceeding an alpha of 0.90, recommended for individual patient analysis [26].

The PedsQL™ Family Impact Module Scales performed as hypothesized utilizing the known-groups method. Where statistically significant differences existed between families with children at the CCH and REACH, REACH families were lower functioning, generally confirming the hypothesis that families whose medically fragile children live in a residential facility are higher functioning than those whose children live in the home.

The present findings have certain limitations. Information on nonparticipants and an accurate response rate were not available, which may limit the generalizability of the findings. The generalizability of the findings is further limited by the small sample size and the selection of medically fragile children with complex chronic medical conditions. Whether the instrument would perform well in groups of children with other chronic health conditions is a matter of empirical inquiry. Given that instrument validation is an iterative process and consistent with this paradigm, the PedsQL™ Family Impact Module will be further field tested in other pediatric chronic health conditions with larger populations of children.

## Conclusion

The study demonstrates the preliminary reliability and validity of the PedsQL™ Family Impact Module, an instrument designed to assess the impact of pediatric chronic health conditions on parents' HRQOL and family functioning. As predicted, families of children with medically fragile conditions who resided in a children's convalescent hospital were higher functioning than families of similar children who resided at home.

## List of Abbreviations

HRQOL Health-Related Quality of Life

PedsQL™ Pediatric Quality of Life Inventory™

## Authors' Contributions

JWV and PD conceptualized the rationale and design of the study. JWV designed the instrument and drafted the manuscript. PED participated in the instrument design and coordination of initial data collection. SAS performed the statistical analysis and participated in study coordination, instrument development, and data collection. TMB participated in study conceptualization and design, instrument development, and data collection. All authors read and approved the final manuscript.

## References

[B1] Varni JW, Seid M, Kurtin PS (1999). Pediatric health-related quality of life measurement technology: A guide for health care decision makers. J Clin Outcomes Manag.

[B2] Matza LS, Swensen AR, Flood EM, Secnik K, Leidy NK (2004). Assessment of health-related quality of life in children: A review of conceptual, methodological, and regulatory issue. Value in Health.

[B3] Varni JW, Wallander JL, Routh DK (1988). Pediatric chronic disabilities. In Handbook of Pediatric Psychology.

[B4] Varni JW, Katz ER, Colegrove R, Dolgin M (1996). Family functioning predictors of adjustment of children with newly diagnosed cancer: A prospective analysis. J Child Psychol Psychiat.

[B5] Thompson RJ, Gustafson KE (1996). Adaptation to Chronic Childhood Illness.

[B6] Wallander JL, Varni JW (1998). Effects of pediatric chronic physical disorders on child and family adjustment. J Child Psychol Psychiat.

[B7] Moos RH, Moos BS (1986). Family Environment Scale Manual.

[B8] Stein RE, Jessop DJ (2003). The Impact on Family Scale revisited: Further psychometric data. J Dev Behav Pediatr.

[B9] Landgraf JM, Abetz L, Ware JE (1996). The CHQ User's Manual.

[B10] Varni JW, Seid M, Rode CA (1999). The PedsQL™: Measurement model for the Pediatric Quality of Life Inventory. Med Care.

[B11] Varni JW, Seid M, Kurtin PS (2001). The PedsQL™ 4.0: Reliability and validity of the Pediatric Quality of Life Inventory™ Version 4.0 Generic Core Scales in healthy and patient populations. Med Care.

[B12] Varni JW, Seid M, Knight TS, Uzark K, Szer IS (2002). The PedsQL™ 4.0 Generic Core Scales: Sensitivity, responsiveness, and impact on clinical decision-making. J Behav Med.

[B13] Varni JW, Burwinkle TM, Seid M, Skarr D (2003). The PedsQL™ 4.0 as a pediatric population health measure: Feasibility, reliability, and validity. Ambul Pediatr.

[B14] Varni JW, Seid M, Knight TS, Burwinkle TM, Brown J, Szer IS (2002). The PedsQL™ in pediatric rheumatology: Reliability, validity, and responsivenessof the Pediatric Quality of Life Inventory™ Generic Core Scales and Rheumatology Module. Arthritis Rheum.

[B15] Varni JW, Burwinkle TM, Katz ER, Meeske K, Dickinson P (2002). The PedsQL™ in pediatric cancer: Reliability and validity of the Pediatric Quality of Life Inventory™ Generic Core Scales, Multidimensional Fatigue Scale, and Cancer Module. Cancer.

[B16] Varni JW, Burwinkle TM, Jacobs JR, Gottschalk M, Kaufman F, Jones KL (2003). The PedsQL™ in Type 1 and Type 2 diabetes: Reliability and validity of the Pediatric Quality of Life Inventory™ Generic Core Scales and Type 1 Diabetes Module. Diabetes Care.

[B17] Varni JW, Burwinkle TM, Rapoff MA, Kamps JL, Olson N (2004). The PedsQL™ in pediatric asthma: Reliability and validity of the Pediatric Quality of Life Inventory™ Generic Core Scales and Asthma Module. J Behav Med.

[B18] Uzark K, Jones K, Burwinkle TM, Varni JW (2003). The Pediatric Quality of Life Inventory™ in children with heart disease. Prog Pediatr Cardiol.

[B19] Varni JW, Burwinkle TM, Szer IS The PedsQL™ Multidimensional Fatigue Scale in pediatric rheumatology: Reliability and validity. J Rheumatol.

[B20] Varni JW, Quiggins DJL, Ayala GX (2000). Development of the Pediatric Hematology/Oncology Parent Satisfaction survey. Child Health Care.

[B21] Varni JW, Burwinkle TM, Dickinson P, Sherman SA, Dixon P, Ervice JA, Leyden PA, Sadler BL (2004). Evaluation of the built environment at a Children's Convalescent Hospital: Development of the PedsQL™ Parent and Staff Satisfaction Measures for pediatric health care facilities. J Dev Behav Peds.

[B22] Fairclough DL (2002). Design and Analysis of Quality of Life Studies in Clinical Trials: Interdisciplinary Statistics.

[B23] Ware JE (1993). SF-36 Health Survey: Manual and Interpretation Guide.

[B24] Fairclough DL, Cella DF (1996). Functional Assessment of Cancer Therapy (FACT-G): Non-response to individual questions. Qual Life Res.

[B25] Cronbach LJ (1951). Coefficient alpha and the internal structure of tests. Psychometrika.

[B26] Nunnally JC, Bernstein IR (1994). Psychometric Theory.

[B27] Pedhazur EJ, Schmelkin LP (1991). Measurement, Design, and Analysis: An Integrated Approach.

[B28] McHorney CA, Ware JE, Raczek AE (1993). The MOS 36-item short-form health survey (SF-36): II. Psychometric and clinical tests of validity in measuring physical and mental health constructs. Med Care.

[B29] McHorney CA, Ware JE, Rogers W, Raczek AE, Lu JFR (1992). The validity and relative precision of MOS short- and long-form health status scales and Dartmouth COOP charts: Results from the Medical Outcomes Study. Med Care.

[B30] Cohen J (1988). Statistical Power Analysis for the Behavioral Sciences.

